# CX3CR1: a potential microglia-specific PET imaging target in Alzheimer’s and Parkinson’s diseases

**DOI:** 10.3389/fphar.2025.1678159

**Published:** 2025-12-04

**Authors:** Hongzhi Yang, Yanli Wang, Yulong Xu, Changning Wang

**Affiliations:** 1 Department of Radiology, Brigham and Women’s Hospital, Harvard Medical School, Boston, MA, United States; 2 Athinoula A. Martinos Center for Biomedical Imaging, Department of Radiology, Massachusetts General Hospital, Harvard Medical School, Charlestown, MA, United States

**Keywords:** microglia, CX3CR1, Alzheimer’s disease, Parkinson’s disease, positron emission tomography tracers

## Abstract

Microglia are the resident immune cells of the central nervous system (CNS), playing a crucial role in maintaining brain homeostasis and mediating neuroimmune responses. The chemokine receptor CX3CR1, predominantly expressed on microglia, regulates microglial function via interactions with its neuronal ligand CX3CL1. The CX3CR1-CX3CL1 signaling exhibits complex, context-dependent roles in neurodegenerative diseases. In Alzheimer’s disease (AD) and Parkinson’s disease (PD) animal models, CX3CR1 deficiency shows paradoxical outcomes, attenuating or exacerbating amyloid-β (Aβ) and tau pathologies in AD, while consistently worsening α-synuclein-induced neurodegeneration in PD. Although CX3CR1 emerges as a promising therapeutic and diagnostic target, its complex role in microglial dynamics remains incompletely understood. Positron emission tomography (PET) imaging provides a powerful, noninvasive method for investigating biological processes *in vivo*. There is an urgent need to develop and validate new PET tracers targeting microglial CX3CR1 in the CNS, further offering new opportunities for the diagnosis and treatment of neuroinflammation-associated neurodegenerative diseases.

## 1 Introduction

**FIGURE 1:**
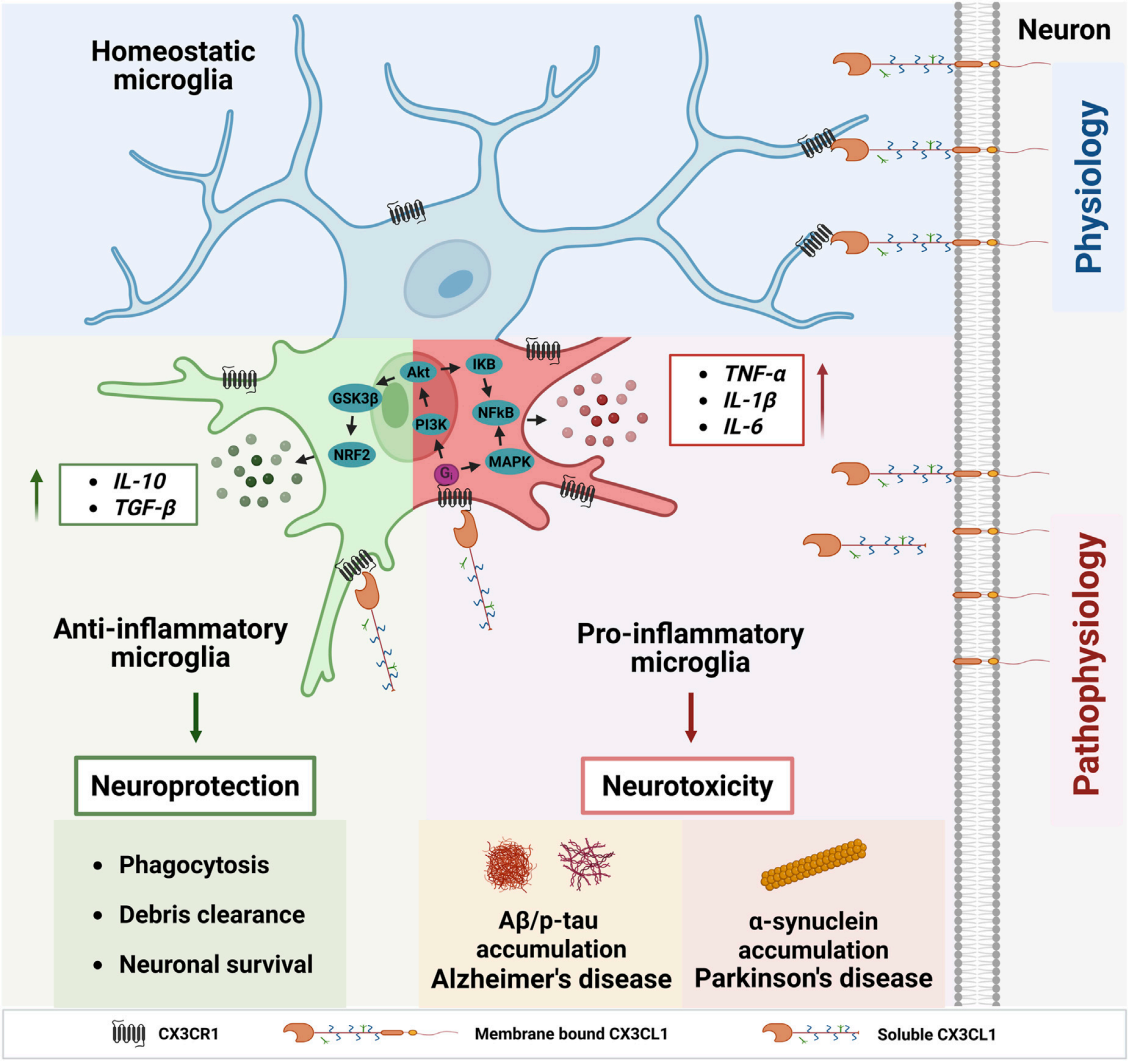
Effects of CX3CR1-CX3CL1 signaling on microglial function. Both membrane-bound and soluble forms of CX3CL1 interact with CX3CR1, triggering G protein-coupled signaling cascades that regulate intracellular responses in microglia. Under physiological conditions, CX3CR1-CX3CL1 signaling suppresses pro-inflammatory cytokine production and maintains microglia in a homeostatic state. In pathological conditions, during the early stages of disease, CX3CR1-CX3CL1 activation exerts neuroprotective effects via the PI3K/Akt/NRF2 pathway, enhancing microglial phagocytosis and anti-inflammatory activity to support neuronal survival. As disease progresses, persistent neuroinflammation induces microglial hyperactivation, and CX3CR1-CX3CL1 signaling shifts toward the PI3K/Akt/NF-κB and MAPK/NF-κB pathways, promoting proinflammatory cytokine release, neuronal dysfunction, and death. These processes collectively exacerbate neurodegeneration in Alzheimer’s and Parkinson’s diseases. *Figure created with BioRender*.

Microglia are the resident immune cells in the CNS, responsible for regulating innate immunity and participating in adaptive immune responses (Garden and Möller, 2006). They originate from the early yolk sac and migrate into the CNS during the early embryonic development stage (around E9.5 and E10.5) (Ginhoux et al., 2010). Under physiological conditions, macroglia exhibit a highly ramified morphology in their homeostatic state. The branched structure enables them to sensitively monitor changes in the CNS microenvironment and maintain brain homeostasis by phagocytosing neuronal synapses, apoptotic cells, and cellular debris, while releasing trophic factors that support neuronal growth and survival (Bachiller et al., 2018). However, under pathological conditions, microglia rapidly adopt hypertrophic morphology characterized by an enlarged cell body and shortened cellular processes. As sentinels, microglia modulate the immune response to minimize potential CNS damage and promote tissue repair (Sadeghdoust et al., 2024). Since neuroinflammation plays a crucial role in the development and progression of neurodegenerative diseases, microglia have gained significant attention for mediating CNS immune responses, and their dysfunctional activation worsens disease progression (Figure 1).

The activation states of microglia are dynamic and context-dependent. They thus cannot be adequately studied through *in vitro* isolation or culturing cells, transcriptomic analysis, or biopsy sampling alone (Jain et al., 2020). Under these conditions, microglia may lose much of their distinctiveness and become prototype macrophages (Wolf et al., 2013). Given that microglia are highly sensitive cells that undergo rapid morphological and functional changes *in vitro*, *in vivo* microglial reporter mouse models have been developed to better study their behavior in the intact CNS environment (Gosselin et al., 2017). The CX3CR1-GFP knock-in mice (in which cDNA encoding green fluorescent protein (GFP) replaces one Cx3cr1 gene allele) are popular experimental models for studying microglial function of AD and PD (Guedes et al., 2018). However, there is an unmet need for noninvasive techniques that enable longitudinal *in vivo* visualization of neuroimmune responses, especially in humans. Positron emission tomography (PET) has been widely applied in both research and clinical settings to study biological processes in living subjects. It is an indispensable tool for early disease detection and diagnosis, real-time therapy monitoring, and patient stratification in clinical trials (Jain et al., 2020).

Microglial receptors play a crucial role in receiving external stimuli, regulating their function, and transmitting signals between cells. Some specific receptor-ligand interaction signaling axes play a key role in the pathogenesis of neurodegenerative diseases (Guedes et al., 2018; Dadwal and Heneka, 2024; Fu et al., 2025). Among them, CX3C chemokine receptor 1 (CX3CR1) stands out as a key molecule, specifically and predominantly expressed on microglia, and recognized by its sole endogenous ligand, CX3CL1 (Mizoue et al., 1999), which is primarily expressed on neurons. The CX3CR1-CX3CL1 axis plays a vital role in modulating microglial function within the CNS (Figure 1) (Dadwal and Heneka, 2024). Recently, overviews of the CX3CR1-CX3CL1 axis in neurodegenerative diseases (Subbarayan et al., 2022; Fu et al., 2025; Vida et al., 2025), drug and PET tracers targeting G protein-coupled receptors (GPCRs) (Pissarek, 2020; Sun et al., 2020; Wang et al., 2025) have been published. From the perspective of CX3CR1, this review provides a brief overview of its basic biology and the dynamics of microglial CX3CR1 in pathological conditions, as observed in mouse models of AD and PD. It discusses its potential as a microglia-specific therapeutic and imaging target in neurodegenerative diseases.

## 2 Biological and structural basis of CX3CR1

Chemokine receptors (CCRs), a subfamily of class A GPCRs, share a conserved seven-transmembrane (TM1-TM7) architecture and dynamic extracellular and intracellular loops (ECLs and ICLs) that enable ligand specificity and signal transduction (Wang et al., 2025). CX3CR1 (Combadiere et al., 1998), a conventional Gαi-coupled receptor and the sole member of the CX3C subfamily, features three amino acids separating two conserved cysteines (Wolf et al., 2013). The membrane-bound form of fractalkine functions as an adhesion molecule that maintains microglia in a quiescent state, whereas the soluble form acts as a chemoattractant, promoting microglial migration toward sites of injury or inflammation (Chidambaram et al., 2020). CX3CL1 binds to CX3CR1, activating intracellular signaling pathways such as PI3K/Akt, NRF2, and NF-κB via G-protein coupling. Under physiological conditions, CX3CR1-CX3CL1 signaling suppresses microglial activation, thereby maintaining a homeostatic phenotype. Under pathological conditions, chronic inflammation driven by the accumulation of Aβ, p-tau, and α-synuclein alters CX3CR1 expression, disrupting signaling balance and modulating microglial activation in a context-dependent manner, leading to both pro- and anti-inflammatory responses. Specifically, CX3CR1-CX3CL1 signaling exerts neuroprotective effects by activating the PI3K/Akt/NRF2 pathway to enhance microglial phagocytic and anti-inflammatory capacity, while exerting neurotoxicity through PI3K/Akt/NF-κB or MAPK/NF-κB to promote the release of the pro-inflammatory cytokines (Eugenín et al., 2023) (Figure 1).

Recent cryo-EM structures of CX3CR1-Gi_1_ complexes in ligand-free and CX3CL1-bound states have elucidated (Lu et al., 2022). CX3CL1 engages CX3CR1 via a two-site binding mode; the globular domain binds the extracellular region (CRS1), while the N-terminal “hook” inserts into the transmembrane core (CRS2). The short β-hairpin loop of ECL2 limits contact with CX3CL1, contributing to the receptor’s unique recognition pattern. Upon binding to soluble or membrane-anchored CX3CL1 (Eugenín et al., 2023), CX3CR1 couples to Gi and transduces extracellular signals via conformational rearrangements. The Gi_1_-coupled structure exhibits a smaller outward movement of TM6, resulting in a narrower G-protein pocket, compensated by outward shifts of TM7 and helix VIII. The unique Gαi coupling conformation establishes extensive interactions among helices I-VIII and ICL2/ICL3, while cholesterol molecules further stabilize this compact active state by maintaining proximity between TM3 and TM6. These structural insights into ligand recognition and receptor activation provide a molecular framework to advance modern drug discovery by enabling the structure-based design of selective CX3CR1 allosteric modulators (Wang et al., 2025).

## 3 CX3CR1 in neurodegenerative diseases

CX3CR1 signaling (CX3CR1/CX3CL1 axis) plays a crucial role in regulating microglial morphology and function in response to neuroinflammation. This signaling pathway exhibits dual effects in neurological pathologies, exerting either neuroprotective or neurotoxic outcomes, depending on the variant microenvironment in the brain and stage of the disease (Luo et al., 2019; Suresh et al., 2021; Subbarayan et al., 2022; Zhao et al., 2023). CX3CR1 signaling confers neuroprotection by suppressing microglial hyperactivation and limiting the release of neurotoxic pro-inflammatory cytokines (e.g., TNF-α, IL-1β), thereby controlling CX3CR1 protein levels and maintaining microglial homeostasis. Conversely, disruptions in CX3CR1 signaling (e.g., CX3CR1 deficiency) lead to excessive microglial activation, heightened inflammatory responses, neuronal loss, and worsening of disease progression. As a result, the specific role of microglial CX3CR1 in neurodegenerative diseases remains controversial and warrants further investigation. This section provides a brief review of the complex roles of CX3CR1 in AD and PD.

### 3.1 CX3CR1 in Alzheimer’s disease

AD is characterized by the extracellular deposition of Aβ and the intracellular aggregation of hyperphosphorylated tau protein into neurofibrillary tangles (NFTs). In parallel, microglial activation-related neuroinflammation is a key contributor to the initiation and progression of AD pathology. Meanwhile, Single-nucleus RNA sequencing (snRNA-seq) studies have revealed distinct clusters of activated microglia associated with Aβ, NFTs, and inflammatory signaling pathways in AD (Nguyen et al., 2020; Gerrits et al., 2021). In human and 5xFAD mice frontal cortical samples, microglial CX3CR1 expression was increased in advanced AD, with CX3CR1 mRNA significantly upregulated at intermediate and advanced stages, indicating CX3CR1 as a potential therapeutic target in disease progression (González-Prieto et al., 2021). The expression of CX3CR1 also increased in the hippocampus of advanced AD patients (Bolós et al., 2017), and upregulation of CX3CR1 gene expression was observed in the hippocampus and frontal cortex of middle-aged 5xFAD mice (Liu et al., 2025). However, *in vivo* studies of CX3CR1 disruption remain challenging, with conflicting outcomes reported across transgenic AD mouse models (Table 1).

**TABLE 1:** Alzheimer’s disease mouse model for investigation of microglial CX3CR1.

Mouse models	Results	Putative mechanism	References
APPPS1; Cx3cr1^^−/−^^ APPPS1; Cx3cr1^+/−^ R1.40; Cx3cr1^−/−^ R1.40; Cx3cr1^+/−^	Decreased plaque burden and Aβ deposition in a gene dose-dependent manner; No alterations in APP levels or processing	Decreased TNF-α and CCL2; increased IL-1β; enhanced Aβ phagocytosis	Lee et al. (2010)
CRND8; Cx3cr1^−/−^	Reduced Aβ deposition and plaque load; increased microglia proliferation around plaques; No effects on synaptic injury	Increased microglial phagocytosis	Liu et al. (2010)
5xTg-Cx3cr1^−/−^ (3xTg-Cx3cr1^−/−^)	Prevention of neuronal loss; No changes in Aβ levels or phagocytosis activity of microglia	Decreased recruitment of microglia to neurons	Fuhrmann (2010)
hTau-Cx3cr1^−/−^	Enhanced tau phosphorylation and aggregation; increased microglia activation; worsening of behavioral impairment	P38 MAPK activation; Induction of TLR4 and IL-1β signaling	Bhaskar et al., 2010; Maphis et al. (2015)
5xFAD; Cx3cr1^−/−^	Accumulation of oligomeric Aβ; aggravates tau hyperphosphorylation, neuronal loss, synaptic dysregulation, and cognitive impairment	Microglial dysfunction through dampened TGFβ-signaling, ROS metabolism	Puntambekar et al. (2022)
APP^swe^/PSEN1^dE9^: Cx3cr1^+/eGFP^	Little impact on the progression of the pathology; higher Aβ plaque density in females than in age-matched males		Hemonnot-Girard et al. (2021)

CX3CR1 deficiency has been associated with both neuroprotective and neurotoxic effects, depending on whether Aβ or tau pathology predominates (Chen, 2016). Systematic studies by Bruce’s group using Cx3cr1-deficient AD murine models have highlighted the divergent roles of neuron-microglial fractalkine signaling in modulating Aβ and tau pathologies (Bhaskar et al., 2010; Lee et al., 2010; Lee et al., 2014; Maphis et al., 2015; Puntambekar et al., 2022). Their findings suggest that CX3CR1 signaling modulates microglial activation triggered by Aβ deposition and further influences downstream neurotoxicity. In the early stages of disease in the APPPS1 and R1.40 mouse models, both complete and partial Cx3cr1 deficiency resulted in a gene-dose-dependent reduction in fibrillar Aβ burden, suggesting a protective role for CX3CR1 loss in Aβ pathology (Lee et al., 2010). Similarly, CRND8; Cx3cr1^−/−^ mice exhibited lower levels of Aβ and reduced amyloid deposits through selective phagocytosis of protofibrillar Aβ (Liu et al., 2010). Recently, Bruce’s group investigated how CX3CR1-mediated microglial response to Aβ promotes sustained neurotoxicity in the 5xFAD amyloid model (Puntambekar et al., 2022). In 5xFAD; Cx3cr1^−/−^ mice, the absence of CX3CR1 impaired microglial endolysosomal activity, reducing the uptake and degradation of fibrillar Aβ. This led to the accumulation of toxic Aβ species and further promoted a pro-inflammatory, neurodegenerative microglial morphology, characterized by dysregulated reactive oxygen species (ROS) metabolism and chronic microglial activation. Long-term, such dysfunction correlates with widespread neurodegeneration, including tau accumulation, synaptic loss, and disrupted neuronal homeostasis. These findings support the notion that the quality and abundance of extracellular Aβ can influence the extent of microglial dysfunction in the absence of CX3CR1 (Puntambekar et al., 2022).

Since most therapeutic interventions only achieve partial target inhibition, understanding the effects of partial CX3CR1 deficiency in AD models is particularly important (Hickman et al., 2019). Hickman et al. reassessed the impact of Cx3cr1 haploinsufficiency using the APPPS1 transgenic AD mouse model. Their results showed that partial CX3CR1 deficiency led to a significant reduction in Aβ deposition and levels across the early to advanced stages of disease, improved cognitive function, and increased levels of Aβ-degrading enzymes (insulysin and matrix metalloproteinase 9 (MMP9)) in the whole brain. These findings suggest that slight downregulation of CX3CR1 activity might be enough to influence neuron-microglia communication, restore neuronal Aβ-degrading enzymes, and ultimately slow the progression of AD-like pathology in APPPS1 mice (Hickman et al., 2019).

CX3CR1 deficiency has been associated with tau pathology. The hTau; Cx3cr1^−/−^ mice exhibit enhanced microglial activation, and the loss of CX3CR1 accelerated neuronal microtubule-associated protein tau (MAPT) hyperphosphorylation and aggregation, as well as the mice’s behavioral impairments (Bhaskar et al., 2010; Kim et al., 2011). These activated microglia lacking CX3CR1 appear to promote the activation of p38 mitogen-activated protein kinase (MAPK) and MAPT hyperphosphorylation, especially upon exposure to microglia-derived interleukin-1β (IL-1β) (Bhaskar et al., 2010; Maphis et al., 2015; Guedes et al., 2018). In parallel, CX3CR1 signaling contributes to the microglial-mediated killing of neurons harboring intracellular tau, thereby facilitating the release and extracellular spread of tau. Furthermore, extracellular tau competes with CX3CL1 for binding to CX3CR1, promoting microglial uptake of tau. Thereby, CX3CR1 deficiency impairs the microglial phagocytosis of tau (Bolós et al., 2017; Chidambaram et al., 2020; Das et al., 2020; Chidambaram et al., 2024). Notably, in 5xTg or 3xTg models, which exhibit both Aβ and tau pathologies, Cx3cr1 deficiency appears to mitigate neuronal loss (Fuhrmann, 2010; Guedes et al., 2018). Increased Aβ load and plaque burden also affect the seeding and spread of pTau and further worsen memory deficits when pathological human-AD tau is injected into 5xFAD and APP-KI mice (He et al., 2018). Consistent with this, 5xFAD; Cx3cr1^−/−^ mice study showed that Aβ-driven microglial dysfunction aggravates tau hyperphosphorylation (Puntambekar et al., 2022). These observations underscore the need for further investigation using AD models that incorporate both Aβ and tau pathologies better to understand the multifaceted role of CX3CR1 in disease progression (Yokoyama et al., 2022; Zhong M. Z, 2024).

Sex and age are major risk factors in AD, with a higher incidence of the disease in females (Laws et al., 2016; Mielke, 2019; Castro-Aldrete et al., 2023). Guillot-Sestier et al. investigated sex-related differences in microglia in APPPS1 mice and in *postmortem* tissue from AD patients (Guillot-Sestier et al., 2021). They found that upregulation of genotype-related microglial activation, increased rod-shaped microglial morphologies, and decreased microglial phagocytosis of Aβ were observed in female mice and AD patients, compared with age-matched males. Complementarily, Hemonnot-Girard et al. examined the impact of Cx3cr1 haplodeficiency in male and female APP^swe^/PSEN1^dE9^ mice across the early to more advanced stages of AD disease (Hemonnot-Girard et al., 2021). Their studies showed that sex and Cx3cr1 haplodeficiency affect Aβ plaque load in a disease-stage-dependent manner. However, sex and CX3CR1 haplodeficiency had little impact on microgliosis progression and cortical neuroinflammation in APP^swe^/PSEN1^dE9^ mice. Notably, they observed a higher Aβ plaque burden and slower learning abilities in females compared to age-matched males, highlighting a sex-dependent difference in disease pathology. Puntambekar et al. also reported that Cx3cr1 haplodeficiency accelerates plaque deposition with disease progression, exacerbates the accumulation/generation of neurotoxic soluble oligomeric Aβ species, and that female 5xFAD mice displayed significantly increased plaque burdens compared to males (Puntambekar et al., 2022). They also found that microglia from 5xFAD; Cx3cr1^−/−^ mice brains express significantly increased mRNA levels of proinflammatory cytokines (e.g., IL-1β) and increased Apoe mRNA levels in females, indicating that CX3CR1 deficiency dysregulates microglial activation towards a neurotoxic phenotype.

### 3.2 CX3CR1 in Parkinson’s disease

PD is the most prevalent neurodegenerative movement disorder, defined pathologically by the progressive loss of dopaminergic neurons in the substantia nigra pars compacta (SNpc) and the abnormal accumulation of α-synuclein (α-SYN) in Lewy bodies and Lewy neurites (Schonhoff et al., 2023). *Postmortem* analyses of PD brains have consistently revealed robust microglial activation, suggesting a key role for neuroinflammation and microgliosis in disease pathogenesis (McGeer et al., 1988; Langston et al., 1999).

CX3CR1 has been reported to exert neuroprotective effects in α-synucleinopathy models of PD by limiting neurotoxicity and attenuating inflammatory responses (Table 2) (Tristão et al., 2016; Vida et al., 2025). In mouse models expressing human α-synuclein, dopaminergic neuronal loss occurred to a similar extent in both wild-type (Cx3cr1^+/+^) and Cx3cr1-deficient (Cx3cr1^−/−^) mice (Thome et al., 2015; Castro-Sánchez et al., 2018). However, in mice overexpressing the A53T mutant form of α-synuclein (α-SYN^A53T^), a mutation linked to familial PD neurodegeneration, the phenotype was significantly worsened in the absence of CX3CR1. This enhanced pathology was accompanied by elevated microgliosis, upregulation of pro-inflammatory mediators, and increased neuronal vulnerability, highlighting the critical role of CX3CR1 in modulating the microglial response to α-syn-induced stress. These findings underscore the importance of CX3CR1 signaling in regulating microglial phenotype and inflammatory cascades during PD progression, particularly in the context of α-synuclein mutations, such as A53T (Castro-Sánchez et al., 2018). Genetic deletion or functional disruption of Cx3cr1 has been shown to exacerbate dopaminergic neuronal loss, with corresponding increases in pro-inflammatory cytokine production and ROS activity in multiple PD mouse models (Nash et al., 2015; Castro-Sánchez et al., 2018). For example, in the MPTP neurotoxin model, Cx3cr1^−/−^ mice exhibited significantly reduced numbers of tyrosine hydroxylase-immunoreactive (TH-IR) neurons in the SNpc, providing further evidence that CX3CR1 contributes to neuronal survival by suppressing microglia-driven neurotoxicity *in vivo* (Cardona et al., 2006). A study by Wang et al. further demonstrated that CX3CR1 expression is downregulated during the early stages of PD and gradually returns to baseline levels in the later stages in rAAV-hSYN-injected C57BL/6 mice, suggesting dynamic regulation of CX3CR1 during disease progression (Wang et al., 2020). These findings support the potential of CX3CR1 as a biomarker for disease staging and a candidate for therapeutic intervention. In parallel, work from Harms et al. (2018) using AAV-SYN; Cx3cr1^+/GFP^ reporter mice confirmed that resident CNS microglia express high levels of CX3CR1 and actively respond to α-synuclein accumulation. However, their results also suggest that much of the observed microgliosis is driven by peripheral monocyte infiltration, complicating the distinction between intrinsic microglial activation and monocyte recruitment during neuroinflammatory responses (Harms et al., 2018).

**TABLE 2:** Parkinson’s disease mouse model for investigation of microglial CX3CR1.

Mouse models	Results	Putative mechanism	References
AAV2-SYN injection in Cx3cr1^−/−^ mice	No exacerbate α-syn-induced neurodegeneration and pro-inflammatory response	Reduced microglial phagocytosis of α-syn	Thome, Standaert and Harms (2015)
MPTP injection in Cx3cr1^−/−^ mice	MPTP injection exacerbates the loss of tyrosine hydroxylase-immunoreactive (TH-IR) neurons	Increased levels of IL-1β	Cardona et al. (2006)
rAAV9-α-SYN^WT^ injection in Cx3cr1^+/+^ mice rAAV9-α-SYN^A53T^ injection in Cx3cr1^−/−^ mice	No change in the expression of CX3CL1 Expression of α-SYN^A53T^ exacerbated neurodegeneration Enhanced neurodegeneration accompanied by a neuroinflammatory process with increased microgliosis	CX3CR1-deficiency shifts microglial dynamics to a pro-inflammatory phenotype	Castro-Sánchez et al. (2018)

## 4 Development of PET tracers targeting CX3CR1

**FIGURE 2:**
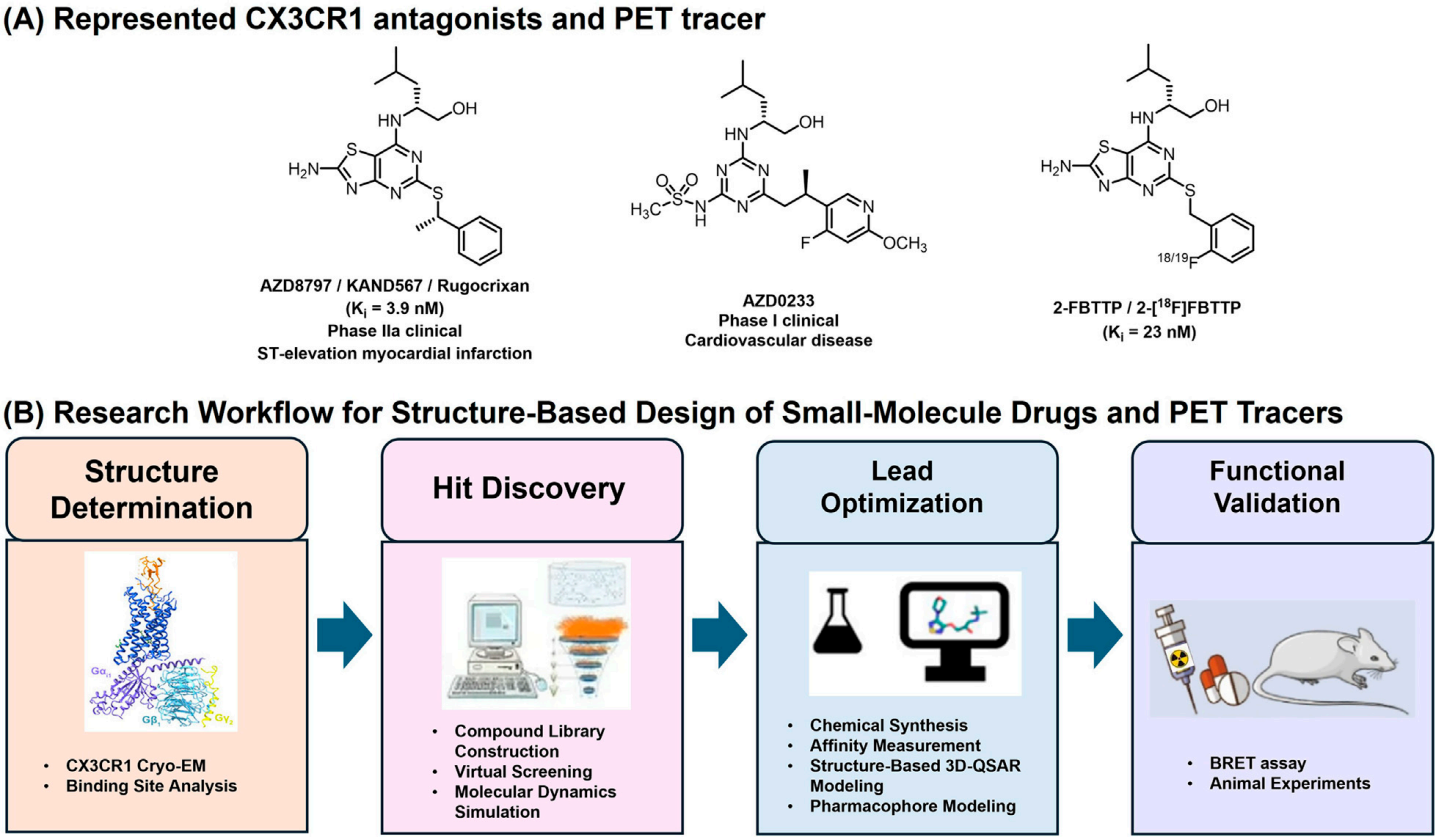
**(A)** Represented CX3CR1 antagonists and PET tracer; **(B)** General Research Workflow for Structure-Based Design of Small-Molecule Drugs and PET Tracers Targeting CX3CR1. The experimental workflow comprises four main parts: cryo-EM CX3CR1 structure determination; structure-based small-molecule Hit discovery; lead optimization and functional validation.

The promiscuity of chemokines and their cognate chemokine receptors (CKRs) has long posed an obstacle to chemokine receptor-based drug development. Although CX3CR1 offers a unique advantage as a potential drug target due to its restricted ligand-receptor pairing with CX3CL1, the development of small-molecule modulators has remained slow and challenging (Lu et al., 2022). To date, only two small-molecule CX3CR1 antagonists have entered clinical evaluation (Figure 2). AZD8797, also known as KAND567 or Rugocrixan, is a potent and selective human CX3CR1 antagonist with high binding affinity (K_i_ = 3.9 nM, K_B_ = 10 nM), 720-fold selectivity *versus* chemokine receptor CXCR2, and favorable physicochemical characteristics suitable for *in vivo* applications (Karlström et al., 2013; Ridderstad Wollberg et al., 2014). It functions as a non-competitive allosteric modulator that interferes with CX3CL1-induced receptor signaling and exhibits biased modulation of downstream G-protein and β-arrestin pathways (Cederblad et al., 2016).

Preclinical studies have demonstrated that AZD8797 (I.P., 80 μg/kg/day) exerts therapeutic effects by inhibiting the CX3CR1-CX3CL1 signaling pathway, thereby suppressing inflammatory responses and promoting tissue repair in CNS inflammatory animal models of spinal cord injury (SCI) (Chen et al., 2020) and eosinophilic meningitis (Lai et al., 2025). For instance, in mice infected with *A. cantonensis*, microglial activation and neuronal degeneration were accompanied by increased p-tau accumulation and elevated CX3CR1 expression in brain tissue; these pathological changes were markedly attenuated following intraperitoneal administration of AZD8797 (Lai et al., 2025). Beyond its effects on neuroinflammation, AZD8797 suppresses CX3CR1-positive monocyte-mediated tumor growth in chronic lymphocytic leukemia (CLL) cells (Zhong W, 2024). It is currently under Phase IIa evaluation in patients with ST-elevation myocardial infarction following percutaneous coronary intervention (Ekinci et al., 2024). Recently, AstraZeneca disclosed a new CX3CR1 antagonist, AZD0233 (Guo et al., 2025). Oral administration of the compound (100 mg/kg/day) can improve cardiac function and reduce fibrotic scar tissue in a mouse model of dilated cardiomyopathy. This compound has advanced into single-ascending dose (SAD) and multiple-ascending dose (MAD) Phase I clinical studies for dilated cardiomyopathy and heart failure. As the development of PET tracers is closely intertwined with advances in drug discovery, 2-[^18^F]FBTTP remains the only reported radiotracer specifically targeting microglial CX3CR1, derived directly from the small-molecule antagonist 2-FBTTP (K_i_ = 23 nM) (Figure 2) (Karlström et al., 2013; Pissarek, 2020). Designed to enable noninvasive visualization of neuroinflammation, 2-[^18^F]FBTTP has demonstrated sufficient blood-brain barrier permeability in healthy mice, supporting its potential for CNS imaging. However, comprehensive biodistribution studies and validation in disease-relevant neuroinflammatory models are still required to establish its translational applicability.

Different target-based small-molecule PET tracers have been developed to image microglial activation. Among these, PET radiotracers targeting the 18 kDa translocator protein (TSPO) have been traditionally and widely used to assess neuroinflammation by visualizing microglial activation in AD, PD, and other neurodegenerative disorders. However, TSPO tracers exhibit several notable limitations, including high nonspecific binding, low brain penetration, and interspecies polymorphic differences that affect binding affinity between rodents and humans (Janssen et al., 2018; Jain et al., 2020; Beaino et al., 2021). Specifically, although TSPO expression increases in activated microglia in mouse models of brain disease, it remains essentially unchanged in human patients, indicating that TSPO-PET signals in humans reflect only the density of inflammatory cells (e.g., microglia/macrophages) rather than the activation state of microglia (Nutma et al., 2023).

To overcome these limitations, alternative microglia-specific imaging targets have been explored (Janssen et al., 2018; Jain et al., 2020; Beaino et al., 2021). Among them, the purinergic P2Y12 receptor (P2Y12R), a G-protein-coupled receptor selectively expressed on homeostatic microglia, has emerged as a promising biomarker for distinguishing microglial phenotypic states during neuroinflammation (Mildner et al., 2017; Pissarek, 2020). Recently, two nicotinate-based PET radiotracers with favorable brain permeability and high binding affinity for P2Y12R have been developed. Yao et al. (2025) reported [^18^F]QTFT (Yao et al., 2025), which enables quantitative imaging of P2Y12 expression on anti-inflammatory microglia across diverse preclinical models and in patients with neuroinflammatory diseases; however, further structural optimization is required to minimize hepatic uptake and off-target binding. Similarly, Joseph et al. described another P2Y12 PET tracer [^18^F]**12** (Joseph et al., 2025), enabling images of P2Y12-positive microglia, although additional modifications are still needed to improve *in vivo* stability and reduce nonspecific binding.

The development of PET tracers targeting CX3CR1 remains at an early stage. The structure-based drug design (SBDD) (Yang et al., 2021; Cao et al., 2024; Cebi et al., 2024) has become an increasingly precise and efficient approach in modern drug discovery, enabling rational optimization based on atomic-level structural insights (Figure 2). Both AZD8797 and AZD0233, representative CX3CR1 antagonists sharing a thiazolopyrimidine scaffold, likely bind to a similar receptor pocket, underscoring the need for new chemotypes to expand the chemical diversity of CX3CR1 ligands. The elucidation of the CX3CR1-Gi_1_ complex provides a structural framework for SBDD, enabling the rational development of allosteric modulators and antagonists guided by the receptor’s distinct extracellular topology, compact intracellular conformation, and cholesterol-binding interfaces. Complementary molecular dynamics (MD) simulations (Goode-Romero and Dominguez, 2022; Piloto et al., 2024) further characterize receptor flexibility, transient pockets, and ligand-binding stability. Together, the integration of cryo-EM structures and MD-guided modeling accelerates rational discovery of CX3CR1-targeted allosteric modulators, facilitating the development of highly specific PET tracers for imaging microglial activation and neuroinflammation in the CNS.

## 5 Discussion

CX3CR1 is a key microglial homeostatic gene that regulates motility, phagocytosis, and inflammatory responses in the CNS. It is selectively expressed on microglia, and the CX3CR1-CX3CL1 axis mediates neuron and microglia communication, maintaining microglial quiescence and immune balance. Notably, CX3CR1 expression is upregulated in the AD patient’s brain, particularly in the frontal cortex and hippocampus, reflecting microglial proliferation and sustained activation in these regions. In contrast, the inconsistent findings observed in experimental models may be partly attributed to CX3CR1 polymorphisms, as human variants (e.g., V249I and T280M) have been associated with altered receptor function and differential susceptibility to AD and PD (Kalinderi et al., 2012; López-López et al., 2018; Meyer, 2025). Although TSPO tracers are widely used, they lack cellular specificity and are strongly influenced by gene polymorphisms (Janssen et al., 2018; Jain et al., 2020; Kreisl et al., 2020; Beaino et al., 2021). Similarly, P2RY12, while highly microglia-specific (Honarpisheh et al., 2020), is markedly downregulated upon activation, and its reduced expression and regional heterogeneity in AD brain tissue further limit its translational potential (Mildner et al., 2017; Walker et al., 2020). Compared with other microglial imaging targets, CX3CR1 offers distinct advantages. It remains microglia-specific and functionally involved in neuron–microglia signaling, and its expression increases throughout disease progression. These characteristics make CX3CR1 a particularly promising target for microglial imaging. However, challenges remain in achieving the optimal balance between binding affinity, selectivity, and blood–brain barrier permeability required for the development of effective PET tracers. Ongoing research aims to develop CX3CR1-targeted PET tracers to noninvasively assess microglial activity and neuroinflammatory dynamics *in vivo*, particularly in AD and PD patients.
